# Semaphorins in Angiogenesis and Autoimmune Diseases: Therapeutic Targets?

**DOI:** 10.3389/fimmu.2020.00346

**Published:** 2020-03-05

**Authors:** Vijaya Iragavarapu-Charyulu, Ewa Wojcikiewicz, Alexandra Urdaneta

**Affiliations:** Department of Biomedical Sciences, Florida Atlantic University, Boca Raton, FL, United States

**Keywords:** semaphorin, neuropilins, plexins, angiogenesis, angiostatic, autoimmunity, MOG, targeted therapy

## Abstract

The axonal guidance molecules, semaphorins, have been described to function both physiologically and pathologically outside of the nervous system. In this review, we focus on the vertebrate semaphorins found in classes 3 through 7 and their roles in vascular development and autoimmune diseases. Recent studies indicate that while some of these vertebrate semaphorins promote angiogenesis, others have an angiostatic function. Since some semaphorins are also expressed by different immune cells and are known to modulate immune responses, they have been implicated in autoimmune disorders such as multiple sclerosis, rheumatoid arthritis, systemic lupus erythematosus and systemic sclerosis. We conclude this review by addressing strategies targeting semaphorins as potential therapeutic agents for angiogenesis and autoimmune diseases.

## Introduction

Semaphorins consist of a large family of conserved proteins originally described as axon guidance molecules during the development of the nervous system. These molecules are now known to be expressed in other adult tissues and function outside of the nervous system (1). Semaphorins since have been discovered to have pleiotropic effects in both health and disease. Semaphorins and their receptors have widespread functional impact physiologically and pathologically as they participate in immune regulation, extracellular matrix remodeling, organogenesis, and angiogenesis (2–4). These molecules therefore play crucial roles in pathophysiology of diseases such as cancer, systemic lupus erythematosus, rheumatoid arthritis, psoriasis, arthritis, proliferative retinopathy, and atherosclerosis among others (5–10). In this review, we discuss semaphorin's structure, receptors, signaling and downstream effects on pathophysiology. We then highlight the roles of semaphorins with respect to angiogenesis and autoimmune disease. We conclude with an emphasis on the role of semaphorins in angiogenesis and autoimmune disease and explore the possibility of targeting semaphorins and their receptors to ameliorate angiogenesis and regulate immune functions.

## Structure, Receptors, and Signaling

**Figure 1:**
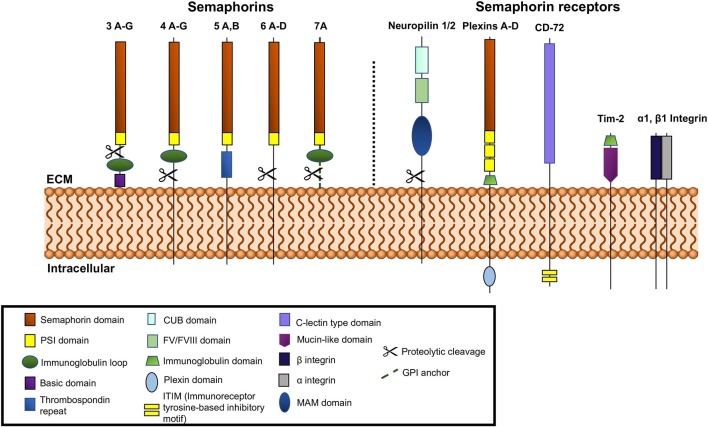
Schematic representation of vertebrate semaphorins and semaphorin receptors. Class 3 is secreted, Classes 4-6 are transmembrane and Class 7 is GPI anchored. Cleavable semaphorins are Sema3, 4, 6, and 7. Receptors for semaphorins are shown on the right side of the figure, neuropilins, plexins, Tim-2, and α1β1 integrin.

The semaphorin family is divided into eight classes, with invertebrate semaphorins belonging to classes 1 and 2, the vertebrate semaphorins being found in classes 3–7, and the viral semaphorins in class 8 (Figure 1). The Sema domain of semaphorins contains approximately 500 amino acids (1). At the carboxy terminus of the Sema domain, all semaphorins also contain a Plexin-semaphorin-integrin (PSI) domain (11). Variations in the C-terminal motifs joining the PSI domain are the key differentiating factor among semaphorins (12). The C-terminus of vertebrate Sema3, 4, and 7 contains an immunoglobulin loop. Sema3 (A-G) contains a basic domain and Sema5 (A-C) contains thrombospondin repeats on their C termini, respectively. Class 3 semaphorins are secreted, classes 4, 5, and 6 are membrane bound and class 7 is the only member that is GPI-anchored (13) (Figure 1). Semaphorins 3, 4, 6, and 7A are susceptible to cleavage by matrix metalloproteinases and adamlysin family proteases (14, 15).

Neuropilins and Plexins serve as semaphorin receptors and are the means through which semaphorins can participate in signal transduction (16–19). Both are transmembrane proteins, with extracellular domains capable of interaction with the semaphorins which can dimerize to mediate their function. Most semaphorins can interact with Plexins directly, while almost all of the class 3 semaphorins (except 3E) bind neuropilins, which form complexes with type A Plexins or Plexin D1. The plexins are required to transduce the signals (13, 16, 20, 21). There are two known neuropilins, -1 and -2 (16, 17) (Figure 1). Both have short intracellular domains. Their interaction with Plexins, which possess a longer intracellular segment, facilitates their involvement in the transduction of pro-angiogenic signals. The extracellular segment of neuropilins is also the site of binding for VEGF, HGF (hepatocyte growth factor), FGF-2, PDGF-B, TGF-β and other ligands (22–24). The Plexins can more robustly participate in signal transduction via their longer intracellular GTP-ase activating domain (GAP domain). The intracellular GAP domain interacts with GTP-ases directly. Plexins are subdivided into classes A, B, C, and D (Figure 1) and interact directly with semaphorins from classes 4, 5, 6, and 7 and Sema3E (25, 26). Plexins A (1–4) and D interact with neuropilins 1 and 2 (25, 26).

Semaphorins are a family of proteins that were initially established as repellent cues in axonal guidance and synapse formation during embryogenesis. It is now known that they not only exert a repulsive effect in axonal guidance but, they can also be attractive axonal cues. Sema3A has repellent effects on neurons while Sema3C is known as an attractant. The other members in this family, Sema3D, Sema3E, and Sema3F have both repellent and chemoattractant effects on axons (27, 28). Semaphorin 4A has been shown to function as a chemoattractant, likely working in concert with other neurotrophic factors to promote neurite outgrowth (29). Sema5A, on the other hand, has been shown to have both attractive and repulsive functions during development (30, 31). Of class semaphorins, Sema6A and Sema6B were shown to have chemorepulsive activity via interaction with Plexin A4 in various models of development and angiogenesis (32–34). Although Sema7A promotes axon growth, chemotropic effect was not evident in a model of rat olfactory bulb explant (35). In addition to their role in axonal guidance, semaphorins also play a role in the periphery in regulating angiogenesis and immune responses.

## Role of Semaphorins in Angiogenesis

Semaphorins play a significant role in vascular development through the promotion or inhibition of angiogenesis. A balance between pro- and anti-angiogenic signals determine the progression of new blood vessel sprouting. Similar to their function in axonal guidance, semaphorins guide endothelial cells toward tube formation for angiogenesis. Pro-angiogenic semaphorins include Sema3C, Sema4A, Sema4D, Sema6D, and Sema7A, while angiostatic semaphorins include Sema3A, Sema3B, Sema3D, Sema3E, and Sema3F (Table 1). Although Sema3C and Sema4A have been shown to have pro-angiogenic activity, they also were reported to function as anti-angiogenic molecules (13, 53) (Table 1).

**Table 1:** The role of semaphorins in mediating angiogenesis and autoimmune diseases.

**Semaphorin**	**Angiogenesis**	**References**
Semaphorin 3A	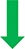	(36–40)
Semaphorin 3B	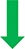	(41, 42)
Semaphorin 3C	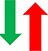	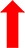 (43, 44) 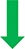 (13, 45, 46)
Semaphorin 3D	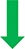	(47)
Semaphorin 3E	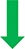	(48, 49)
Semaphorin 3F	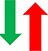	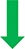 (40, 50) 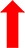 (51)
Semaphorin 4A	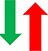	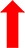 (52) 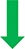 (53) 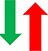 (54)
Semaphorin 4D	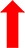	(55, 56)
Semaphorin 5A	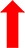	(57, 58)
Semaphorin 6D	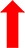	(59)
Semaphorin 7A	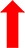	(60–62)

*
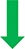
Anti-angiogenic; 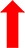
Pro-angiogenic*.

Class 3 semaphorins are for the most part anti-angiogenic. Class 3 semaphorins exert angiogenic effects through interactions with co-receptors neuropilin-1, -2 (NRP-1,−2) and vascular endothelial growth factor (VEGF) receptor family. Semaphorins 3A, 3B, 3D, 3E, and 3F are exclusively anti-angiogenic (Table 1). The anti-angiogenic activity of Sema3A was demonstrated using cultured rat aortic rings. Sema3A inhibited capillary sprouting and it was further shown to inhibit endothelial cell migration (36). Using an oxygen-induced retinopathy mouse model, Yu et al. showed that injection of the intravitreous region with Sema3A reduced neovascularized areas and decreased abnormal vessel growth (37). Acevedo et al. showed that Sema3A interferes with VEGF-induced angiogenesis (38). Recently, in a mouse model of bronchial asthma by Adi et al. have shown that treatment of mice with Sema3A reduced inflammatory cell infiltration in bronchioles and angiogenesis was significantly decreased compared to the untreated controls (39). Sema3A and Sema3F are characterized as anti-angiogenic by competing with VEGF in binding to endothelial cell expressed neuropilins (NRP-1/2), the co-receptors for VEGF family (40). Further, Guttmann-Raviv et al. found that co-expression of Sema3A and Sema3F repel endothelial cells more potently than either one of the semaphorins alone (40). Sema3B also was found to have anti-angiogenic activity via NRP-1/-2 which resulted in the repelling of endothelial cells, induction of apoptosis, and inhibition of tube formation (41). Rolny et al. determined the role of Sema3B in tumor angiogenesis and found a reduction in angiogenesis in mice injected with Sema3B transduced tumor cells (42). Similarly, Sema3D/NRP-1 activity was found to inhibit cell motility and tube formation in endothelial cells (47). In contrast, Sema3E was determined to be anti-angiogenic via Plexin-D1, and not NRP signaling on endothelial cells *in vitro* and *in vivo* (63). Sakurai et al. reported that Sema3E's anti-angiogenic activity can be attributed to its inactivation of R-Ras and stimulation of Arf6 factors which affect integrin activity and inhibit endothelial cell adhesion (63). Other studies have also elucidated Sema3E/Plexin-D1's activity to work as a regulatory mechanism for VEGF-induced angiogenesis by modulating the ratio of endothelial tip and stalk cells (24). Studies with Sema 3E^−/−^ mice revealed the important role that avascular zones generated by Sema3E play in guiding cardiac vessel development (48). Further, in a rat model of ischemic stroke, it was shown that Sema3E/Plexin-D1 signaling inhibited angiogenesis through regulation of endothelial dynamic delta-like 4 molecule (64).

Within class 3 semaphorins, Sema3C is one of the exceptions due to its bifunctional activity as both a pro-angiogenic and anti-angiogenic factor (13, 43, 45, 65). *In vitro* studies showed Sema3C to induce endothelial cell proliferation, adhesion and directional migration (43). However, other studies report Sema3C to be significantly anti-angiogenic (13, 45). Pathologic angiogenesis was shown to be inhibited by Sema3C in an oxygen-induced retinopathy model (45). Further, these authors showed that Sema3C inhibits endothelial tube formation when Human Umbelical Vein Cells were cultured with Sema3C conditioned medium. The anti-angiogenic activity of Sema3C was shown by overexpressing Sema3C in U87 glioblastoma cells and assessing formation of neovasculature in chick chorioallantoic membranes (CAM). Sema3C overexpressing U87 cells did not induce new vessels while control U87 cells had extensive vessels on CAMs (66). Therefore, the effects of this semaphorin may be environment dependent and are ultimately controversial. Sema3F contrary to majority of class 3 semaphorins, was shown to promote extraembryonic angiogenesis via inhibition of Myc-regulated throbospondin 1 in yolk sac epithelial cells (50). In contrast, other studies showed that Sema3F is expressed in the avascular outer region of retina and that it exerts anti-angiogenic effects on the retinal and choroidal capillaries (51).

Within class 4 semaphorins, Sema4D was found to have pro-angiogenic effects. Both soluble and membrane-bound forms of Sema4D have been described as pro-angiogenic by signaling through endothelial receptors, Plexin-B1 and Plexin-B2. Interaction of Sema4D with Plexin-B1 stabilizes vasculature. Sema4D has been shown to have potent angiogenic effects both *in vitro* and *in vivo* by inducing endothelial cell chemotaxis, tube formation, cytoskeletal rearrangements, and vessel growth (55, 56). Increased levels of Sema4D have been correlated with poor prognosis in studies of leukemia and mammary carcinoma (67–69). Interestingly, this semaphorin has been shown to play a role in vasculogenic mimicry in a non-small cell lung cancer model. Xia et al. found that the interaction of Sema4D with PlexinB1 promoted vasculogenic mimicry while inhibition of Sema4D decreased vasculature (70). In contrast to Sema4D, Sema4A was found to have dual activity as both a pro- and anti-angiogenic factor. The pro-angiogenic effect of Sema4A in the context of tumor is indirectly mediated by signaling through Plexin-D1-expressing macrophages, which induce VEGF-A expression and thereby enhance tumor vasculature (52). However, depending on the environment, Sema4A inhibits angiogenesis using the same receptor, Plexin-D1 (53). Therefore, the role of Sema4A in tumors is still controversial.

The only member in class 5 semaphorins reported to have angiogenic activity is Sema5A. This semaphorin has been shown to be necessary for normal cranial vasculature development and be a regulator of angiogenesis by promoting endothelial cell migration and proliferation, while also reducing apoptosis (57, 58).

Among class 6 semaphorins, Sema6D acts by binding to a receptor complex composed of PlexinA1 and either Off Track (OTK) or VEGFR2. Binding of Sema6D to these receptor complexes results in varying effects during cardiac development including, endothelial cell repulsion or attraction, respectively (2). In models of gastric cancer, signaling due to Sema6D and Plexin-A1/VEGFR2 interaction results in effects similar to VEGF binding alone. In addition, Sema6D/Plexin-A1 expression is positively correlated with the expression of VEGFR2, therefore contributing to its angiogenic and tumorigenic properties (59). Poor prognosis of gastric cancer has been correlated with Sema6D expression and increased angiogenesis (59) (Table 1).

Class 7 semaphorins have also been found to have pro-angiogenic effects (Table 1). In particular, Sema7A was determined to mediate angiogenesis through signaling via Plexin-C1 and β1 integrins. Using a corneal neovascularization model, Ghanem et al. showed that Sema7A is expressed in vascularized corneas and that basic fibroblastic growth factor (bFGF) enhances the expression of Sema7A (60). The pro-angiogenic function of Sema7A in promoting intraplaque neovascularization was found to be mediated through β1 integrin and activation of VEGFA/VEGFR2 (61). Tumor angiogenesis is regulated by stromal cells such as macrophages, neutrophils and cancer associated fibroblasts (71). Tumor angiogenesis is affected by infiltration of leukocytes, e.g., tumor associated macrophages (TAMs) (72). Due to its chemotactic effects, Sema7A could attract TAMs which could then regulate angiogenesis in the tumor microenvironment (73). Garcia-Areas et al. delineated the angiogenic role of Sema7A in promoting tumor growth. In this study, it was shown that co-culture of Sema7A with macrophages induces the production of angiogenic chemokines, CCL2, CXCL2/MIP2. Further, implantation of Sema7A gene-silenced mammary tumor cells resulted in decreased *in vivo* tumor angiogenesis compared to the wild type tumors (62). Thus, in the context of tumor, Sema7A could promote angiogenesis in multiple ways. Further, Black et al. revealed a novel role for Sema7A in promoting lymphangiogenesis in breast cancer and reported that loss of Sema7A reduces both tumor cell invasion and activation of β1-integrin receptor (74).

## Role of Semaphorins in Autoimmune Disease

Semaphorins through interaction with their receptors, in addition to playing a role in angiogenesis, regulate immune homeostasis, and tissue inflammation. Neuropilins are important for the initiation of the primary immune response as NRP-1 has been shown to mediate contact between DCs and T cells in the immunologic synapse (75). Autoimmune disorders are characterized by dysregulated immune responses associated with decreased T regulatory cells and overactive responses by B and T cells against self-molecules. T regulatory development is guided by the transcription factor, Foxp3 (76). In a mouse model, it was shown that T_reg_ cells express NRP-1. However, it is important to note NRP-1 is not a marker of human Foxp3 T_reg_ cells (77). The interaction of NRP-1 with immune cell-expressed Sema4A in mice further potentiates T_reg_ cell function (78). Further, peripheral tolerance is also maintained by dendritic cells that could prevent activation of self-reactive cells which can then lead to inhibition of autoimmunity. The receptors expressed at the immunological synapse between dendritic cells (DCs) and T cells can therefore affect the outcome between development of tolerance or autoimmune response (79).

Semaphorins, Sema3A, Sema3E, Sema4A, Sema4D, Sema5A, Sema6D, and Sema7A may be considered as “immune semaphorins” since they are involved in physiological and pathological immune responses (80). Autoimmune diseases, such as systemic lupus erythematosus (SLE), rheumatoid arthritis (RA), multiple sclerosis (MS), and systemic sclerosis or scleroderma (SSc), are characterized by chronic inflammation and subsequent tissue damage resulting from cellular and humoral immune responses to self-antigens. Inflammation affects the expression of semaphorins and their receptors and recent studies show that several members of the semaphorin family are aberrantly expressed in autoimmune disorders (Table 2) (89, 90). In this review, we focus on immune semaphorins as one of the mediators of autoimmune diseases.

**Table 2:** The role of semaphorins in mediating autoimmune diseases.

**Semaphorin**	**Autoimmune disease**	**References**
Semaphorin 3A	Rheumatoid Arthritis (RA), asthma, systemic sclerosis (SSc), Multiple Sclerosis (MS), systemic lupus erythematosus (SLE)	(81, 82)
Semaphorin 3C	RA	(83)
Semaphorin 3E	SSc	(49)
Semaphorin 3F	MS	(84)
Semaphorin 4A	Experimental autoimmune encephalomyelitis (EAE), MS, RA	(85)
Semaphorin 4D	MS/EAE, RA	(85–87)
Semaphorin 5A	RA	(88)
Semaphorin 6D	EAE	(21)
Semaphorin 7A	RA, MS/EAE, SSc, COPD	(85)

**Figure 2:**
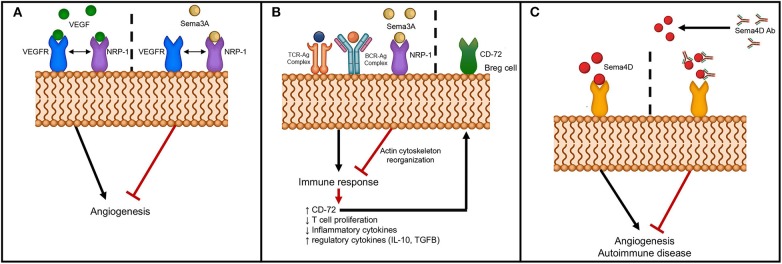
Schematic of semaphorin interaction with receptors to modulate angiogenesis and autoimmunity. **(A)** Sema3A interferes with angiogenesis through binding to NRP-1, the co-receptor for VEGFR; **(B)** Sema3A dampens immune response through binding to NRP-1 with downstream effects on actin cytoskeletal reorganization and upregulation of CD72 on B regulatory cells; and **(C)** Inhibition of angiogenesis and autoimmunity by neutralizing anti-Sema4D antibody.

The secreted class 3 semaphorins modulate immune responses by binding and signaling through neuropilins and their association with Plexins. The members of the semaphorin 3 family that function in pathogenesis of autoimmune diseases are Sema3A, Sema3C, Sema3E, and Sema3F. Sema3A is a potent immunoregulatory molecule and has been shown to suppress the over-activity of T and B lymphocytes (91–93). Activation of naïve T cells requires an immunological synapse with dendritic cells in the secondary lymphoid organs. The immunosuppressive role of Sema3A on T cell proliferation was first described by Lepelletier et al. (94). NRP-1, the Sema3A receptor expressed by activated T cells and DCs., was found to play an important role in forming DC-T cell synapse (75). Lepelletier et al. found that the high levels of Sema3A produced in the later stage of DC-T cells co-cultures inhibited T cell proliferation. Thus, the induced Sema3A expression by both DCs and T cells during the latter part of the immune response could be regulating this response (94). Either neutralization by blocking antibodies or by an antagonist peptide of Sema3A increased T cell proliferation (94). These authors have shown that the immunomodulatory function of Sema3A is mediated by actin cytoskeleton reorganization that has downstream effects on signal transduction (94) (Figure 2B). Solomon et al. have shown that NRP-1 attenuates autoreactivity of myelin oligodendrocyte glycoprotein (MOG)-induced experimental autoimmune encephalitis (EAE) and that lack of NRP-1 aggravates the disease (95). Furthermore, Lepellier et al. have also shown that both Sema3A and Galectin-1 expressed by mesenchymal stem cells inhibit T cell proliferation through NRP-1 binding (96).

Other studies have shown that Sema3A downregulates T cell activation and modulates immune responses through activation of T regulatory cells (81). Further, co-culture of B regulatory cells with Sema3A upregulated expression of CD72 and enhanced the production of immunoregulatory cytokines, IL-10 and TGF-β (97) (Figure 2B). More significantly, culturing of Sema3A with cytosine-phosphodiester-guanine oligodeoxynucleotides (CpG-ODN)-stimulated B cells from SLE patients resulted in decreased TLR-9 expression that could then have an effect on cytokine production profile (98). Several studies have linked pathogenesis of autoimmune diseases to lower Sema3A levels and serum levels were reported to inversely correlate with disease activity of SLE, RA and SSc (81, 97–99) (Table 2). Catalano reported downregulation of Sema3A in T cells from RA patients (91). Further, transient ectopic expression of Sema3A inhibited clinical manifestation of collagen induced arthritis (91). Rezaeepoor et al. found that serum levels of Sema3A and its expression in peripheral blood mononuclear cells were significantly decreased in MS patients compared to normal subjects (100). In contrast, Williams et al. showed an increase in expression of Sema3A at the inflammatory regions from brains of human patients (84). It is possible that Sema3A is involved in the regeneration of oligodendrocytes, and deregulation of Sema3A could impair recruitment of oligodendrocyte precursors preventing repair. T helper cell differentiation and transmigration through the blood brain barrier are also detrimental in mediating pathogenesis of MS. Lack of Sema3A or its receptors resulted in impaired T cell priming and studies show that inhibiting immune cell migration prevents MS relapse (85). These studies indicate that Sema3A downregulates autoimmune disease by suppressing both B and T cell activity (93). The role of Sema3A in SSc is unclear, while some studies have shown reduced expression of Sema3A in serum and in regulatory T cells, others did not detect any differences in expression levels between SSc patients and normal individuals (81, 82).

Another member of class 3 semaphorins, Sema3C, has been implicated in RA (Table 2). Miller et al. showed that synovial tissue samples from RA patients were positive for Sema3C and synovial macrophages and fibroblasts were found to express Sema3C by immunofluorescence (83). In contrast to decreased Sema3A levels in SSc, elevated levels of Sema3E were found in both serum and skin from SSc patients (49) (Table 2). Impaired angiogenic response following tissue ischemia and hypoxia is an important feature of SSc (101). Thus, the anti-angiogenic effect of the Sema3E and Plexin-D1 interaction results in the dysregulation of vascular tone control and may contribute to pathogenesis of SSc. The last member of the semaphorin 3 family implicated in autoimmunity is Sema3F. The transcripts of Sema3F were upregulated in the brains of MS patients and in experimental models of demyelination (84, 102). Increased Sema3F expression was associated with glial cell infiltrates in the inflammatory lesions (84). These authors suggested Sema3F expression influences oligodendrocyte precursor cell recruitment that could promote re-myelination.

Class 4 semaphorins also play a role in autoimmune diseases (Table 2). The effects of Sema4 members are mediated by binding to class B Plexins, Tim-2, CD72, NRP-1, and NRP-2 among others (4, 103–106) (Figure 1). Additionally, Sema4A and Sema4D may be cleaved producing soluble forms (21). Both of these semaphorins have been associated with pathology of RA. Levels of Sema4A and Sema4D are increased in serum and synovial fluid of RA patients (87, 107). These elevated levels have been positively correlated with serum levels of inflammatory cytokines, TNF-α and IL-6 (107). Sema4A is expressed in activated T cells and DCs and plays a critical role in the immune system as it is involved in antigen-specific T helper cell responses (108, 109). Pathogenesis of MS is mediated in part by dysregulated helper T cells. Since Sema4A plays a role in T helper cell differentiation, it has been associated with pathogenesis of MS. Further, the use of anti-Sema4A (anti-CD100) monoclonal antibodies significantly suppressed the development of EAE (108). Others have shown that mice lacking Sema4A have diminished TH1 responses; this suggests that these mice may be less prone to EAE, which is mediated by TH1 cells (109). Another member of class 4 semaphorins which is implicated in autoimmune disease is Sema4D. While Sema4D is expressed at low levels in B cells, it is expressed at higher levels in T cells. The interaction of T cell expressed Sema4D with CD72 on DCs augments T cell activation (110, 111) (Table 2). By binding to Plexin B1 and CD72, Sema4D promotes activation of B cells to induce antibody production and antigen specific T cells (86, 110, 112). Okuno et al. demonstrated attenuation of MOG-specific EAE development by adoptive transfer of MOG-specific T cells into Plexin-B1 deficient mice, which indicates the role of the Sema4D-Plexin B1 interaction in pathogenesis of EAE (88).

Among class 5 semaphorins, Sema5A is the only member thus far that has been associated with autoimmune disease (Table 2). High levels of secreted Sema5A were found in circulation of patients with RA (113). Further, treatment of primary T cells and NK cells with soluble form of recombinant Sema5A resulted in increased proliferation and secretion of proinflammatory TH1 and TH17 cytokines (113).

A class 6 semaphorin, Sema6D, is expressed in lymphoid populations including T, B and NK cells. O'Connor et al. studied the regulation of T cells by Sema6D, the stimulation of which resulted in enhanced Sema6D expression (114). Sema6D interacts with Plexin A1 and TREM-1/DAP12 complex to activate T cells and generate antigen specific T cells (85). In mice lacking Plexin A1, production of antigen-specific T cells is defective. Therefore, these mice are less prone to developing EAE (21). These studies suggest a potential role for Sema6D in the development of MS.

Semaphorin 7A, an immune semaphorin, plays an important role in regulating innate immune cells. In the immune system, Sema7A is expressed by activated T lymphocytes and stimulates not only monocytes, but also macrophages to produce proinflammatory cytokines. Sema7A was found to induce the production of proinflammatory cytokines through monocytes (73) and activated T-cells (4) (Figure 1). By binding to α1β1 integrin in both monocytes (115) and T cells, Sema7A activates the MAP kinase pathway (43, 115). This finding departs from the notion that semaphorins signal only through Plexins and neuropilins, the traditional semaphorin receptors. As a GPI-anchored protein, Sema7A is recruited to lipid rafts that accumulate at the immunological synapse between T cells and macrophages. Direct immunization of Sema7A-deficient mice with MOG peptide and adoptive transfer of antigen-specific Sema7A-deficient T cells do not induce T-cell-mediated immune responses (115). Sema7A-knockout mice resist the development of inflammation after hapten-induced contact hypersensitivity (85). In human studies, Sema7A has been shown to be involved in chronic inflammatory diseases like chronic obstructive pulmonary disease (COPD) (116) and RA (117) (Table 2).

In addition to its role in the immune response, Sema7A, the only GPI-anchored semaphorin, functions as a chemoattractant and stimulates neuronal migration. Other semaphorins such as Sema4D (118), Sema4C (119), and Sema6A (120) have also been shown to promote neuronal migration. More importantly, Sema7A promotes dendricity not only in axons (35), but also in melanocytes (121), osteoclasts (122), activated T-cells (4), and monocytes (73). Expression of Sema7A has also been associated with fibrosis, inflammation and immune modulation, and is shown to play a role in RA, MS and SSc (123–125) (Table 2).

Sema7A is cleaved off the membrane by ADAM-17 (15). In patients with RA, the elevated levels of soluble Sema7A in both serum and synovial fluid have been correlated with disease severity (99, 125). Xie et al. showed that soluble Sema7A activates TH1 cells resulting in increased production of the inflammatory cytokines IL-6 and IL-17 that could contribute to pathogenesis of RA (125). Costa et al. studied the expression of Sema7A in lesions of MS patients and correlated the levels to the severity of the inflammation in the lesions (126). Using an EAE mouse model, Gutierrez-Franco et al. elucidated the role of Sema7A in MS by comparing demyelination or cell death in Sema7A deficient mice with wild type mice. Mice deficient in Sema7A had impaired inflammatory cellular infiltrates into the central nervous system and reduced demyelination compared to wild type littermates (124). Further, decreased circulating levels of Sema7A have been associated with patients with SLE compared to healthy controls (99).

Sema7A is also an important regulator of tissue remodeling by inducing fibrosis (116, 127). A pulmonary fibrosis study showed that expression of Sema7A and its receptors, Plexin C1 and α1β1 integrins, are induced by TGF-β1 contributing to TGF-β1-derived fibrosis and tissue remodeling mediated by the PI3K/AKT pathway (116). Similarly, recent studies found Sema7A in astrocytes and, accumulation of Sema7A in fibrotic tissue following spinal cord injury via activation the PI3K/AKT pathway (127). Sema7A knockout mice crossed with TGF-β1 overexpressing transgenic mice exhibited decreased severity in lung fibrosis compared to TGF-β1 overexpressing transgenic control mice (123). Collagen-producing fibrocytes and B cells expressing Sema7A contribute to pulmonary fibrosis and thus could lead to SSc (123).

## Targeting Semaphorins to Control Angiogenesis and Autoimmune Diseases

Numerous studies have implicated semaphorins as therapeutic targets for angiogenesis and autoimmune diseases. However, the strategies depend on various factors. For example, semaphorins can either promote or inhibit angiogenesis depending on the receptor they engage with, whether it is a transmembrane or a secreted molecule, and which signaling pathways are activated. Further, semaphorin signaling is modulated by the receptor and co-receptor complex. Thus, different combinations of receptor complexes can affect signaling pathways to result in altered cytokine production, cell proliferation and migration and, ultimately, causing either angiogenesis or angiostasis. Similarly, dysregulated immune responses contributing to autoimmune disorders are also affected by transmembrane *vs*. secreted semaphorins, the receptors engaged and the signaling pathways activated. All of these factors must be considered when designing therapeutic strategies. So, what are some of the possible strategies to control angiogenesis and/or autoimmune diseases mediated by semaphorins? Some strategies include the use of soluble semaphorins, small molecules or blocking antibodies to inhibit signaling, and antagonist peptides to inhibit sema-receptor complexes. Addressed in this review are soluble semaphorins and antibodies to ameliorate angiogenesis and autoimmune disease.

Studies show that Class 3 semaphorins have anti-angiogenic activity (128–130). Sema3A, -C, and E have all been shown to be anti-angiogenic. Thus, class 3 semaphorins have been used as “physiological vascular normalizing agents” for anti-cancer therapy and thereby, aid in enhancing the efficacy and overcoming acquired resistance to anti-angiogenic therapies (130). *In vitro* studies show that migration of endothelial cells cultured in the presence of angiogenic inducers is inhibited by Sema3A and Sema3F (38, 129, 131). In mouse models of cancer, systemic delivery of Sema3A impaired angiogenesis and metastasis (128). A possible mechanism by which Sema3A inhibits angiogenesis is by competing for neuropilin, a co-receptor for VEGF (Figure 2A). Anti-angiogenic activity of Sema3E is mediated through Plexin D1 to regulate endothelial cells and development of vasculature (132). Sema3E-plexin D1 interaction inhibits angiogenesis by suppressing the VEGF signaling pathway (133). It may be postulated that semphorins such as Sema3A or Sema3E can be used as anti-angiogenic agents to block the pro-angiogenic activity of semaphorins such as Sema4A or Sema4D. Using an oxygen-induced retinopathy model, Yang et al. found that local administration of Sema3C inhibits pathological angiogenesis (45). Further, both tumor angiogenesis and lymphangiogenesis were inhibited by the stabilized form of Sema3C (65). In a glioblastoma model, ectopic expression of Sema3D or Sema3E reduced tumor growth (134). Using a RipTag2 pancreatic tumor model, Tamagnone et al. showed inhibition of tumor angiogenesis by administering Sema3E via an Alzet pump delivery system (135). These studies indicate that semaphorins may be used as therapeutic agents to regulate angiogenesis. However, a potential problem with the use of semaphorins as treatment agents for angiogenesis are the possible side effects, e.g., those caused by suppressing the VEGF pathway by Sema3E/Plexin D1.

In terms of its possible use in treating autoimmune diseases, Sema3A is a viable candidate as it has been shown to have immunoregulatory activities on both innate and adaptive immunity (136). Treatment with Sema3A and subsequent binding to NRP-1 suppresses the immune response and also enhances B regulatory cells by upregulating CD72 (137) (Figure 2B). In a mouse model of RA, overexpression of Sema3A partially attenuated disease progression (91). Further, treatment of mice with Sema3A was beneficial in that it reduced lupus nephritis (136). Behar et al. showed that increased Sema3A expression on B regulatory cells and that addition of Sema3A to activated B cells resulted in downregulation of TLR-9 expression (136). Sema3A could therefore be added to the arsenal of treatment options for MS, SLE and other autoimmune disorders.

Antibodies provide an attractive treatment option to directly target specific molecules to block the action of semaphorins and thus, reduce angiogenesis or suppress autoimmune diseases. However, there are difficulties in targeting semaphorins due to: (1) the conserved Sema domain in semaphorins and Plexins; (2) redundancy in semaphorins; and (3) receptors that bind to molecules other than semaphorins. Despite these difficulties, antibodies have been designed and manufactured providing positive results. Semaphorins interact with their receptors, neuropilins, and Plexins, to mediate the downstream effects. Studies have shown that targeting neuropilins, Plexins, or semaphorins with specific antibodies results in decreased angiogenesis. Semaphorin 4D blocking antibody was used to assess the level of inhibition of angiogenesis *in vitro* and *in vivo*. Reduced vessel counts were observed in mice that received anti-sema4D antibodies indicating reduced angiogenesis (56) (Figure 2C). Kong et al. using anti-NRP-1 peptide in both *in vitro* and *in vivo* studies found suppression of VEGF-induced angiogenesis and experimental arthritis (138).

Blocking of semaphorins and preventing interaction with their receptors provides a unique strategy to inhibit autoimmune diseases. It is known that CD4 T cells proliferate and differentiate into TH1 or TH2 cells when presented with an antigen by DCs. TH1 cells not only promote cell-mediated immunity but are involved in development of autoimmune disease. NRP-1 is one of the molecules involved in stabilization of DC-T cell interaction (75). Incubation of either T cells or DCs with NRP-1 antibodies reduced T cell proliferation. This could have implications in developing treatment options for autoimmune diseases. Using an *in vivo* experimental model of axotomy of the rat optic nerve, Shirvan et al. demonstrated that injecting anti-Sema3A antibodies inhibited retinal ganglion cell loss and neuronal protection from degeneration was observed (139). These studies led to the use of the semaphorin antibodies and peptides as possible treatment options for immune mediated diseases. Administration of anti-Sema4A monoclonal antibodies during MOG-induced EAE blocked the development of EAE (108). Other studies have shown that use of neutralizing anti-Sema4D antibodies in treating EAE and RA decreased disease severity (140). Fisher et al. determined that anti-Sema4D antibodies ameliorate collagen-induced arthritis and reduced inflammation in a collagen-induced arthritis model (140). In other studies, administration of anti-Sema4D reduced the severity of RA (107), and using anti-Sema7A antibodies, Xie et al. reported inhibition of collagen induced arthritis (125) (Figure 2C). Using antibodies as therapeutics, one must be cognizant of off-target effects on vasculature, vascularized organs, brain, and spinal cord. In addition to antibodies peptides targeting semaphorin receptors may be an alternative strategy to ameliorate autoimmune diseases. A recent study used Plexin-A1 antagonist to counteract the anti-migratory effect of Sema3A in oligodendrocytes. It was shown that blocking PlexinA1, the receptor of Sema3A enhanced myelin content and thus locomotor activity in an *in vivo* model of EAE (141).

## Conclusion

Considering that the field of study of semaphorins is relatively new, tremendous progress has been made in understanding their roles in various diseases affected by angiogenesis and autoimmune reactivities. Designing effective strategies to reduce pathogenicity associated with these molecules is crucial. In this review, we discussed the role of immune semaphorins, Sema3A, 3C, 3E, 3F, 4A, 4D, 5A, 6D, and 7A in angiogenesis and autoimmune diseases. We then highlighted the inhibition of semaphorins or their receptors in ameliorating angiogenesis and autoimmune diseases.

## Author Contributions

VI-C selected the topic and wrote the introduction, autoimmune disease, and therapeutic approach and conclusion sections. EW wrote the semaphorin structure and signaling and prepared the Table. AU wrote the angiogenesis section and prepared Figures 1, 2. All of the authors critically read and edited the manuscript.

### Conflict of Interest

The authors declare that the research was conducted in the absence of any commercial or financial relationships that could be construed as a potential conflict of interest.
